# Cardiovascular Implications of Lipoprotein(a) and its Genetic Variants: A Critical Review From the Middle East

**DOI:** 10.1016/j.jacasi.2025.04.012

**Published:** 2025-07-01

**Authors:** Ayman El-Menyar, Naushad A. Khan, Wael Al Mahmeed, Jassim Al Suwaidi, Hassan Al-Thani

**Affiliations:** a Clinical Research, Vascular Surgery, Hamad Medical Corporation, Doha, Qatar; b Clinical Medicine, Weill Cornell Medicine, Doha, Qatar; c Heart and Vascular Institute, Cleveland Clinic Abu Dhabi, Abu Dhabi, United Arab Emirates; d Cardiology Department, Heart Hospital, HMC, Doha, Qatar; e Vascular Surgery, HMC, Doha, Qatar

**Keywords:** cardio-genomics, cardiovascular diseases, dyslipidemia, lipid, lipoprotein(a), Middle Eastern population, single nucleotide polymorphism

## Abstract

Dyslipidemia, a significant risk factor for cardiovascular diseases (CVDs), is prevalent in the Middle East (ME) countries. With a variable prevalence, elevated lipoprotein (a) [Lp(a)] is the most widespread monogenic dyslipidemic disorder. Genetic studies have established Lp(a) as a heritable and independent risk factor for CVD. This discovery has shifted the perception of Lp(a) and the *LPA* gene from mere biomarkers of atherosclerotic risk to a viable target for therapeutic intervention. Significant differences in serum Lp(a) levels have been observed across racial and ethnic groups, with few independent genetic variants affecting Lp(a) levels outside the *LPA* gene region. Data specific to the ME and Arab populations remains scarce. ME populations exhibit genetic diversity and higher consanguinity rates, which may uniquely influence Lp(a) distribution and associated variations. This review examines the genetic and observational factors that shape Lp(a) levels and their role in CVD risk, focusing on ME populations.

## Highlights

• The burden of CVDs and dyslipidemia in the developing countries, including the ME, is high.
• ME populations exhibit genetic diversity with higher consanguinity rates, which may influence Lp(a) distribution and *LPA* polymorphisms.
• Despite the Lp(a) structure being close to that of LDL-C, it is more atherogenic.
• The resources for high-quality, in-depth research on Lp(a) and *LPA* are limited in the ME.

Cardiovascular diseases (CVDs) are the predominant cause of mortality globally, accounting for ≈30% of deaths each year. The burden of CVDs in developing countries, including the Middle East (ME), is high, in which coronary artery disease (CAD) is a leading cause of death.^1^ Dyslipidemia is a significant risk factor for CAD worldwide. In the ME, the prevalence of high low-density lipoprotein cholesterol (LDL-C) is 32%.^2^ Notably, Arab patients in the ME tend to experience myocardial infarction (MI) at a younger age than their counterparts of Western descent, highlighting a distinct difference in the onset of major adverse cardiovascular events (MACE).^3^

According to the World Health Organization (WHO) projections, CVD-related mortality in Arab countries is expected to surpass that of other geographic regions. Data indicates that Arab countries, such as the United Arab Emirates, Kuwait, Jordan, Qatar, Lebanon, and Saudi Arabia, have high age-standardized mortality rates for CVDs.^4^ Data from 1990 to 2021^5^ showed that the age-standardized prevalence rate (ASPR) of CVD in the United Arab Emirates was between 10,490.2 and 11,668.2 cases per 100,000 people. At the same time, a higher ASPR of CVD was shown in Syria (1,334.6-1,657 cases per 100,000 people). Saudi Arabia showed an increase of around 14.9% in the CVD ASPR in 2021. Similarly, Libya showed the most significant relative increase of approximately 6.1% in the CVD age-standardized incidence rate.

This contrasts with data from Tunisia, which experienced a significant reduction in the CVD ASPR (from 1,155.8 to 1,138.7/100,000). Egypt showed the highest rate in the age-standardized rate of CVD-related deaths in the same period, which increased from 8,633.6 to 9,918.3/100,000 people.^5^ Moreover, countries like Iraq and Yemen showed higher values for deaths, whereas Kuwait and Qatar showed lower values. The high incidence of metabolic syndrome, diabetes mellitus (DM), dyslipidemia, and consanguinity in the Arab ME contributes to a unique lipid profile characterized by elevated low-density lipoprotein (LDL) levels.^6^

The distinct dyslipidemic patterns in the ME are attributed to various factors, including genetic predisposition and lifestyle choices. The Arab population is highly diverse, comprising a major pan-ethnic group. This region has historically been an intersection of different cultures, leading to significant genetic heterogeneity.^7^ This genetic diversity may substantially impact the prevalence and characteristics of CVDs.^3^ For instance, metabolic syndrome is prevalent among populations in this region.^8^ Furthermore, familial hypercholesterolemia and a high rate of DM exacerbate the cardiovascular (CV) risks.^9^

Lipoprotein(a) [Lp(a)] is a plasma lipoprotein that consists of LDL particles covalently linked to apolipoprotein (a) [Apo(a)]. Notably, Lp(a) is structurally similar to LDL-C, but it is considered more atherogenic caused by the presence of Apo(a), which may exacerbate atherothrombosis by promoting vascular inflammation and exhibiting antifibrinolytic properties that inhibit the plasminogen activation.^10^ Lp(a) also functions as an acute-phase reactant, and its levels can be acutely influenced by cytokine release during the inflammatory response to MI.^11^ However, most Lp(a) genetic research has focused on populations in Asia, Europe, and North America, which possess different genetic vulnerabilities and environmental exposures. Consequently, the relevance of identified Lp(a) genetic loci in these studies may not apply to ME ethnic groups. Elevated Lp(a) and its LPA gene are common monogenic risk factors for CAD, valvular, and vascular disorders.^12^, ^13^, ^14^, ^15^ Despite high cardiovascular risk and substantial genetic diversity among ME populations, this region remains significantly underrepresented in global Lp(a) studies. Significant gaps persist, including lack of population-specific SNV data, absence of large-scale cohort studies, limited genetic screening, and no regionally validated polygenic risk scores (Central Illustration). Notably, no studies from the ME addressed the non-CAD implications for Lp(a) (Table 1).^16^, ^17^, ^18^, ^19^, ^20^, ^21^, ^22^, ^23^, ^24^, ^25^, ^26^, ^27^, ^28^, ^29^, ^30^, ^31^, ^32^, ^33^, ^34^, ^35^

**Central Illustration:**
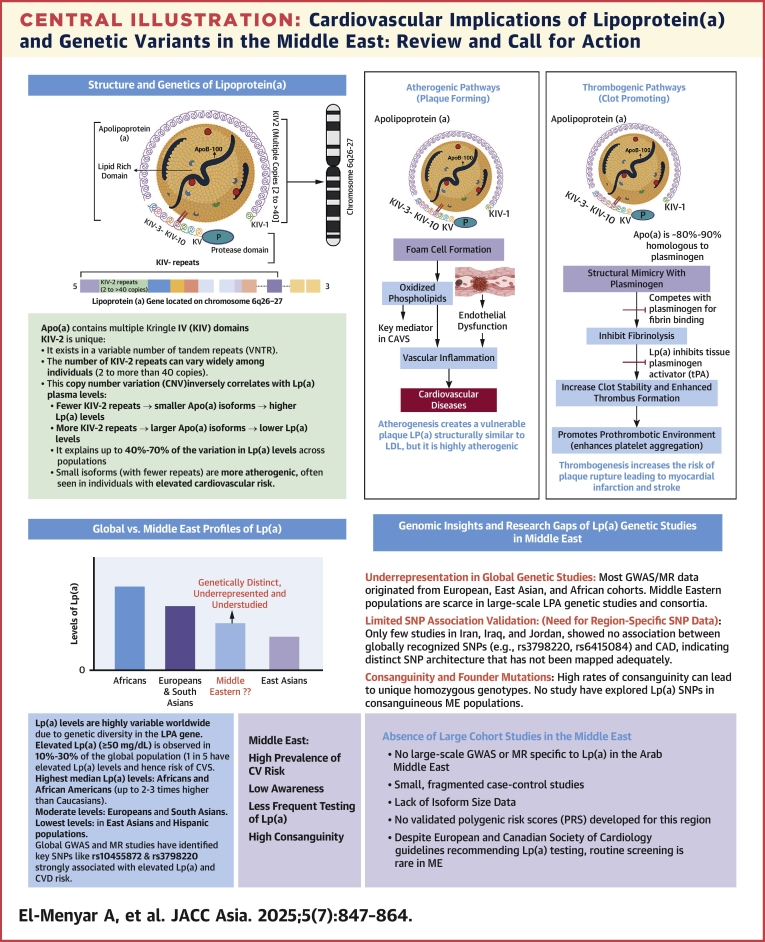
Cardiovascular Implications of Lipoprotein(a) and Genetic Variants in the Middle East: Review and Call for Action Lipoprotein(a) [Lp(a)] is a genetically determined lipoprotein strongly associated with increased risk of atherosclerotic cardiovascular diseases, including coronary artery disease, calcific aortic valve stenosis, and stroke. Its plasma levels are regulated by the *LPA* gene, particularly through kringle IV-2 (K-IV-2) copy number variation; fewer repeats result in smaller Apo(a) isoforms, and markedly higher Lp(a) concentrations. Lp(a) promotes cardiovascular pathology through proatherogenic mechanisms, and prothrombotic effects. Despite high cardiovascular risk and genetic diversity in Middle Eastern populations, this region remains underrepresented in global Lp(a) studies. Significant gaps persist, including lack of population-specific SNV data, absence of large-scale cohorts, limited genetic screening, and no regionally validated polygenic risk scores in the Middle East (ME). CAVS: Calcific aortic stenosis; GWAS/MR: genome-wide association study/ Mendelian randomization; SNVs = Single nucleotideVariation. Figure created in BioRender.

**Table 1:** Atherosclerotic and Nonatherosclerotic Cardiovascular Disorders Associated With Lp(a) Level and *LPA* Variants

Condition	Comments
General population^21^^,^^22^^,^^24^^,^^25^	• Elevated Lp(a) is the common monogenic dyslipidemic disorder with variable prevalence. • Lp(a) values are higher in women than in men and in African Americans compared with other ethnic populations. • 1 of 5 subjects has elevated Lp(a) (>50 mg/dL or 100 nmol/L). • The rs10455872 and rs3798220 SNVs in *LPA* gene explained 25% and 8% of plasma Lp(a) variation. • Lp(a) values >50 mg/dL are found in 10%-30% of the population globally, thus this prevalence is 3 times higher than that for DM.
Middle Eastern populations^18^, ^19^, ^20^	In **KSA**, the Lp(a) levels in the study population were 35 nmol/L (mean) and 50 nmol/L (median). Women showed have higher Lp(a) concentrations than males. In the **United Arab Emirates**, around 1 in 6 patients tested for Lp(a) higher Lp(a), and CVD was prevalent in one-third of patients with high Lp(a). In **Lebanon** and **Jordan** study, despite ongoing statin therapy, goal LDL-C levels were not achieved in less than one-of patients, including approximately 67% of those considered to be at very high risk, however, Lp(a) levels were not tested in this study.
ASCVD^21^^,^^25^	• ASCVD events rate is a 2- to 3-fold higher, when Lp(a) is elevated at any level of LDL-C. • High levels of Lp(a) are generally associated with acute coronary syndrome and large AMI, rather than stable angina. • Patients with elevated Lp(a) values had significantly more recurrent AMI and MACE. • A 100 nmol/L increase in Lp(a) is associated with an 8.0% increased risk of composite MACE, and 18.6% increased risk of coronary revascularization during follow-up.
HF^16^	According to the MR analysis, increased Lp(a) value was significantly associated with increased risk of HF
AF^16^^,^^28^	• An increase in Lp(a) was associated with an increased risk of AF in MR studies. • Each 50 nmol/L (23 mg/dL) increase in Lp(a) was associated with an increased risk of incident AF using measured Lp(a) and genetically predicted Lp(a).
AS^29^	• For rs10455872, the OR for AS was greatest in subjects age 55-64 y and declined with age. • Each rs10455872 risk allele was associated with AS that was diagnosed 0.71 years earlier.
AS^23^	Lp(a) levels >100 mg/dL were significantly associated with risk for severe degenerative AS and subsequent AV incompetence, regardless of the baseline severity of AS.
AS undergoing TAVR^30^	• Lp(a) ≥60 mg/dL were detected in 22.5% of patients. • Elevated Lp(a) might not exert a significant effect on calcification levels or all-cause mortality in AS patients undergoing TAVR.
AS^31^	• The minor alleles of both rs10455872 and rs3798220 *LPA* gene loci were associated with higher risk for aortic valve calcification and stenosis. • High-Lp(a) individuals showed faster AVS progression, by a mean difference of 0.09 m/s/y and a higher risk of death.
AS^17^	• A SNV (rs10455872) in *LPA*, the lipoprotein(a) (Lp(a)) gene is significantly associated with the presence of aortic valve calcification and AS in European cohorts.
Carotid stenosis^21^^,^^22^	• Elevated Lp(a) level is a risk factor for carotid stenosis and ischemic stroke. • The stroke risk (ischemic type) is increasing as high as 10-fold in those with Lp(a) >90th percentile.
AAA^21^^,^^33^	• Patients with AAA show significantly higher Lp(a) levels compared with control subjects. • AAA aortic thrombosis, and aortic dissection were associated with elevated Lp(a).
AD^34^	• Patients with AD had higher median Lp(a) level than non-AD (152.50 vs 81.75 mg/L). • Lp(a) was associated with AD in a multivariate logistic regression analysis with OR of 8.0.
PAD^21^^,^^35^	• Increased Lp(a) with PAD severity, determined by lower ankle-brachial index (ABI) values. • High Lp(a) levels are highly correlated with PAD in people with and without DM.
PAD^24^^,^^35^	• There is a U-shaped distribution of Lp(a) across ABI categories, hence, the high ABI may reflect medial artery calcification that mask underlying atherosclerosis. • The association between elevated Lp(a) and PAD was strongest in Hispanic American men and women and not in the European, African, and Chinese Americans.

AAA = abdominal aortic aneurysm; AD = aortic dissection; AF = atrial fibrillation; AMI = acute myocardial infarction; Apo(a) = apolipoprotein (a); AS = aortic stenosis; ASCVD = atherosclerotic cardiovascular disease; CAD = coronary artery disease; CVD = cardiovascular disease; GWAS = genome-wide association studies; K = Kringles; LDL-C = low-density lipoprotein; Lp(a) = lipoprotein(a); ME = Middle East; MR = Mendelian randomization; PAD = peripheral arterial disease; TAVR = transcatheter aortic valve replacement.

This review focuses on the ME region (18 countries), of which 13 countries are part of the Arab world. The area includes countries from 3 continents: north Africa, West Asia, and Europe (Cyprus and Türkiye). Egypt, Türkiye, and Iran are the most populous countries in the ME. Moreover, Arabs represent the leading ethnic group in the region, followed by Turks and Persians. Furthermore, ME has a diversity of Gross Domestic Product (per capita) (GDP) and literacy rates. While most countries are low income, a few are rapidly growing high income. Therefore, the resources for high-quality, in-depth research are variable and limited, particularly in personalized, genetic, and precision medicine. Most lipid research has limited clinical trials and sample size, and the quality assessment was lacking. This review explores the key observational and genetic factors determining plasma Lp(a) levels and focuses on the sources of inter-individual variability concerning ME countries.

## Structural and Genetic Architecture of Lp(a)

**Figure 1:**
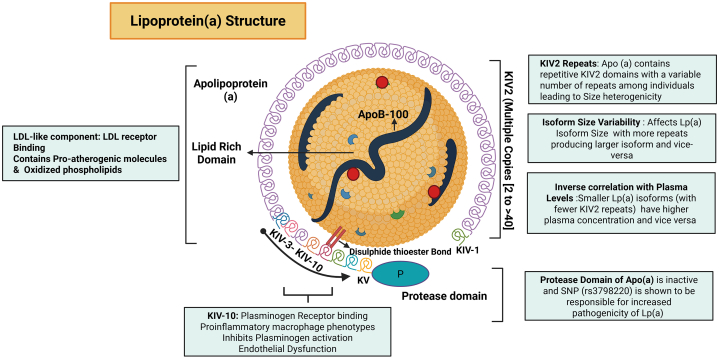
Overview of Lipoprotein (a): Structure The molecular structure of lipoprotein (a) [Lp(a)], has two key components: an LDL-like core containing apolipoprotein B-100 (ApoB-100), and a covalently bound apolipoprotein(a) [Apo(a)]. The Apo (a) domain contains multiple kringle IV type 2 (KIV-2) repeats, with variable copy numbers (2 to >40), resulting in isoform size heterogeneity. Smaller isoforms, associated with fewer KIV-2 repeats, are inversely correlated with higher plasma Lp(a) levels. The structure also includes kringle IV types 3–10, kringle V (KV), and an enzymatically inactive protease domain, which is implicated in Lp(a) pathogenicity due to SNV rs3798220. Functionally, the Lp(a) complex promotes atherothrombosis via LDL receptor binding, oxidized phospholipid transport, and inhibition of fibrinolysis. K-IV-10 binds to plasminogen receptors, leading to pro-inflammatory macrophage phenotypes and endothelial dysfunction, further contributing to cardiovascular risk. LDL = Low-density lipoprotein; SNV = Single nucleotide Variation. Figure created in BioRender.

Lp(a) is a complex group of lipoproteins synthesized in the liver independent of the LDL-C, with a half-life of 3 to 4 days.^36^ Recent advances in genetic research have brought Lp(a) to the forefront of dyslipidemia research.^37^ The distinguishing feature of Lp(a) is its outer shell, which consists of an LDL-like particle (ApoB-100) covalently attached to a large glycoprotein known as Apo(a)^38^ (Figure 1). Apo(a) is of variable size, which substantially influences the overall structure and function of Lp(a). Apo(a) is characterized by multiple repeating units called kringles (K), which are looped protein structures. In humans, Apo(a) contains 2 types of K (V and IV). The K-IV component is exciting because it exists in 10 subtypes, with the K-IV-2 subtype present in a genetically variable number of repeats. This variability leads to significant differences in the size of Apo(a) among individuals, which in turn can affect Lp(a) levels and CV risk.

The core of Lp(a) is similar to that of LDL, consisting of a lipid-rich domain embedded with Apol B100 (ApoB-100), which is linked to Apo(a) via a disulfide bond. ApoB-100 is known for its atherogenic properties, which can contribute to the development of vascular plaques. Additionally, Apo(a) includes a nonfunctional protease domain, which is a remnant of its evolutionary history. This domain originates from the gene for plasminogen, a key thrombolytic enzyme in the body's fibrinolytic system. Thus, Lp(a) might interfere with the normal fibrinolysis process, promote platelet activation, and alter fibrin network structure.

**Figure 2:**
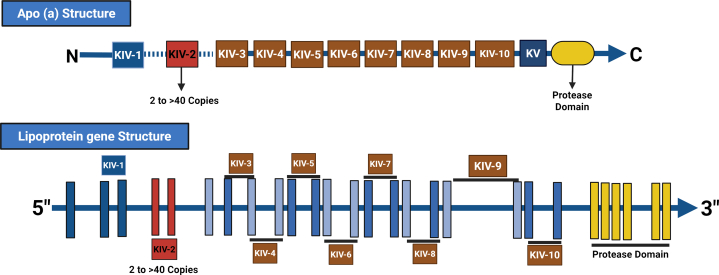
The structural organization of Apolipoprotein (a) [Apo(a)], and its Encoding Gene, *LPA* The upper panel depicts the Apo(a) protein, comprising multiple kringle IV (KIV) domains, including a highly variable kringle IV type 2 (KIV-2) repeat (2 to >40 copies), which directly influences Lp(a) plasma concentration. The C-terminal region includes a kringle V (KV) domain and an inactive protease domain. The lower panel shows the *LPA* gene structure on chromosome 6q26–27, highlighting exon arrangements encoding the KIV types. Notably, the KIV-2 repeat expansion is shown within the gene sequence, reflecting its critical role in apo(a) isoform size heterogeneity. This structural variation is a key genetic determinant of Lp(a)-associated cardiovascular risk, particularly in populations with high inter-individual variability such as those in the Middle East (ME). Figure created in BioRender.

The *LPA* gene (Figure 2) is a product of evolutionary processes originating from the plasminogen gene (PLG), which plays a crucial role in the ability to break down blood clots. The *LPA* gene retains about 70% of its sequence similarity to PLG, highlighting its close evolutionary relationship. One of the most notable changes in the LPA gene is the expansion and diversification of the K-IV domain. K-IV-2 is particularly interesting because it exists in multiple copies within the *LPA* gene. The number of K-IV-2 repeats varies between individuals, contributing to the diversity of Lp(a) levels. The Lp(a) concentration is predominantly controlled by the *LPA* gene locus, which encodes Apo(a).

The presence of Apo B-100 in Lp(a) facilitates its binding to LDL receptors, promoting cholesterol accumulation in the arterial intima. This accumulation triggers an inflammatory response characterized by the recruitment of macrophages, which ingest lipid particles and transform into foam cells, a hallmark of atherosclerotic plaques.^39^ Elevated Lp(a) levels correlate with increased vascular inflammation and enhanced trafficking of peripheral blood mononuclear cells to the arterial wall, further exacerbating atherosclerosis compared with individuals with normal Lp(a) levels.^10^

Moreover, Lp(a) is rich in oxidized phospholipids (OxPLs), which significantly amplify its atherogenic properties.^40^ OxPLs are known to be proinflammatory, facilitating the recruitment of inflammatory cells to the vascular endothelium and accelerating the formation and progression of atherosclerotic plaques.^41^ OxPLs are also key mediators in the development of the atherosclerosis process and calcific aortic valve stenosis (CAVS).^42^ The colocalization of OxPLs with Apo(a)-Lp(a) in arterial lesions contributes to endothelial dysfunction, lipid deposition, inflammation, and osteogenic differentiation, which can lead to vessel calcification.^43^ Lp(a) also disrupts endothelial cell function by reducing nitric oxide production and increasing oxidative stress, which worsens vascular inflammation. These alterations contribute to the destabilization and rupture of atherosclerotic plaques, significantly increasing the risk of acute CV events.^44^

## Cardiovascular Implications of Lp(a)

Lp(a) has been the subject of extensive research, particularly about its role in atherosclerosis, degenerative valvular diseases, and heart failure (HF).^27^ Large-scale genome-wide association studies (GWAS) and Mendelian randomization (MR) data have identified numerous genetic allelic variants of Lp(a) gene linked to CVDs. Moreover, epidemiological, GWAS, and MR data have provided a clear role for Lp(a) as an independent and highly heritable risk factor for the development of atherosclerotic cardiovascular disease (ASCVD) and mortality.^13^^,^^36^^,^^45^ GWAS have identified significant single-nucleotide variations (SNVs) at the *LPA* locus on chromosome 6q26–27, specifically rs3798220 and rs10455872, which are strongly and independently associated with both Lp(a) levels and CAD risk.^46^, ^47^, ^48^ Moreover, it has been shown that the heritability of Lp(a) levels is remarkably high, estimated to be between 70% and 90%, primarily influenced by SNVs in the *LPA* gene and copy number variations in the K-IV type 2 domain.^37^^,^^49^ The heritability of Lp(a) levels underscores its significance as a genetic risk factor for CAD.

There exists considerable interindividual variability in plasma Lp(a) concentrations, primarily attributable to genetic variations in the *LPA* gene. In the general population, Lp(a) levels can range from <1 mg/dL (2.5 nmol/L) to over 1,000 mg/dL, with notable differences observed across various ethnic groups. Elevated Lp(a) levels have been implicated in the development of atherosclerotic plaques; a threshold value of 50 mg/dL has been established, although the risk may begin to escalate at levels above 30 mg/dL.^50^ The definition of high Lp(a) levels varies because of several influencing factors, including the type of assay and measurement units used, the ancestral background, and the clinical characteristics of the study cohort. These variables introduce complexities in defining consistent thresholds for clinical application, complicating the development of standardized Lp(a) cutoffs for assessing CV risk across diverse populations.^37^

Although large-scale GWAS have identified numerous genetic alterations linked to CVD, their findings remain inconclusive. This is caused by 2 main challenges: First, CVD comprises a range of complex, multifactorial events involving various pathways and phenotypes, with multiple genes, each contributing minimally to overall risk. Second, study designs vary widely regarding sample size, clinical endpoints, patient types, and ethnic representation, with limited research focusing on CAD-related allelic variants in the Arab ME.

Despite global advancements in understanding Lp(a), data from the ME population remains limited, thus presenting a significant gap in understanding the role of Lp(a) in this region. The genetic diversity and high prevalence of consanguinity may influence the distribution of Lp(a) levels and their associated allelic variants. There are few studies using a small number of cases from Qatar on the genetics of dyslipidemia in patients with CAD. Recently, researchers in Qatar examined the burden of rare variants in lipid-related genes in CAD using whole genome sequencing. However, these studies did not include an assessment of Lp(a) and *LPA* gene.^51^^,^^52^

The EPIC-Norfolk study highlighted the association between Lp(a) levels and CVD risk across ten European countries (Denmark, France, Germany, Greece, Italy, the Netherlands, Norway, Spain, Sweden, and the United Kingdom). These ethnicities make the study results not completely generalizable to ME populations. Therefore, comprehensive studies in ME cohorts remain needed.^53^ Moreover, the genetic landscape of the ME is characterized by a high degree of variability, which may affect the expression and function of Lp(a). Additionally, the interaction between Lp(a) levels and common metabolic disorders prevalent in the region, such as DM, remains poorly understood, which further complicates the risk assessments.^54^

## Subclinical Atherosclerosis and Lp(a) in ME Populations

Early detection of atherosclerosis using imaging modalities such as coronary artery calcium (CAC) scoring, carotid intima-media thickness, coronary artery plaque, and perivascular fat attenuation index. Individuals from South Korea with higher Lp(a) levels were significantly associated with subclinical atherosclerosis detected by coronary CTA, even after adjustment for clinical and laboratory variables.^55^ A recent meta-analysis using coronary CTA in asymptomatic individuals showed that elevated Lp(a) increased the odds of CAC >0 (OR: 1.31), CAC ≥100 (OR: 1.29), and CAC progression (OR: 1.43). For each increment of 1 mg/dL in Lp(a) there was a 1% in the odds of CAC >0 (OR: 1.01). A study performed in an ME country (Qatar) using coronary CTA showed a high prevalence of obstructive CAD (≥50% luminal stenosis) in a middle-aged cohort. There were no significant differences in coronary CTA findings between Qataris and South Asians after adjustment for clinical risk factors. However, this study lacked information on the Lp(a).^56^ Another study was designed to evaluate the correlation between intima-media thickness of the common carotid artery (CCAIMT), carotid plaque, and absolute cardiovascular risk in a multiethnic population.^57^ The study included 21 countries in Asia, Africa, the Middle East (717 of 2,634), and Latin America. CCAIMT, presence of plaque, and cardiovascular risk factors were assessed for every individual. In Asia, where the subjects were at lower risk, the mean CCAIMT was 4% lower than in Africa/Middle East and Latin America.^57^

## Thrombogenic and Atherogenic Properties of Lp(a)

The structural similarity between Apo(a) and plasminogen is critical for understanding the role of Lp(a) in atherothrombosis.^58^ However, unlike plasminogen, which facilitates fibrinolysis, Apo(a) lacks this activity and instead acts as a competitive inhibitor, obstructing plasminogen's access to fibrin and endothelial surfaces. This inhibition is significant because it reduces plasmin generation, leading to diminished fibrinolysis and promoting clot persistence.^59^ The competitive nature of Apo(a) against plasminogen underscores its role in creating a prothrombotic status and increasing the risk of plaque rupture.^60^ Moreover, Lp(a) exacerbates the risk of CV events by interfering with tissue plasminogen activator (tPA) binding to plasminogen.^61^ This dual action of Lp(a), atherogenic and thrombogenic, highlights its role as a significant CV risk factor.^62^

Elevated Lp(a) levels are increasingly acknowledged as a critical risk factor for ASCVD. Individuals with high Lp(a) have a 2- to 3-fold increase in ASCVD event rates, independent of LDL-C levels, with risk increasing proportionally with rising Lp(a) levels.^63^ In contrast to traditional CV risk factors that often emerge later in life, Lp(a) levels are genetically determined and influenced from birth, like conditions such as familial hypercholesterolemia.^64^^,^^65^

Globally, high Lp(a) levels, defined as ≥50 mg/dL, affect approximately 10% to 30% of the population.^63^ This prevalence is notably higher than that of DM, suggesting that the disease burden associated with elevated Lp(a) may surpass that of DM. The National Heart, Lung, and Blood Institute defines the atherothrombotic range for Lp(a) as ≥30 to 50 mg/dL, indicating that the actual prevalence of elevated Lp(a) could be even greater if lower thresholds are considered.^14^ Given the substantial global prevalence of elevated Lp(a) and its significant association with ASCVD, there is a pressing need for increased awareness and potential therapeutic strategies targeting Lp(a) levels in high-risk populations. Some studies indicate a two-fold increase in CV risk even after adjusting for traditional CV risk factors in patients with elevated Lp(a).^66^ Moreover, the role of Lp(a) in CAD is further complicated by its interaction with other lipid parameters and inflammatory markers.

## GWAS and Mendelian Randomization Integration

**Figure 3:**
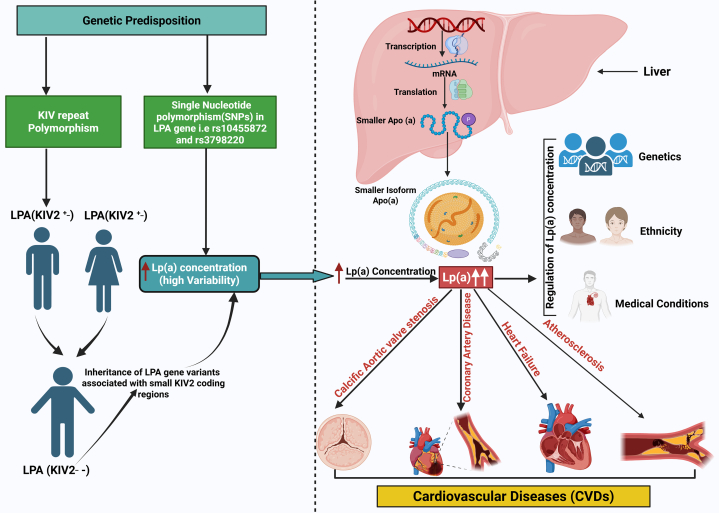
Genetic Regulation of Lipoprotein (a) Concentration, and its Role in CVDs Key genetic determinants influencing plasma lipoprotein(a) [Lp(a)] levels include kringle IV type 2 (KIV-2) repeat variation, specific single nucleotide variations (SNVs), and ancestral genetic diversity; all of which play a central role in modulating Lp(a) concentrations and associated cardiovascular risk. Fewer KIV-2 repeats result in smaller isoforms and elevated Lp(a) levels. Genetic variations, including SNVs such as rs10455872 and rs143431368, further drive inter-individual variation. Significant differences in Lp(a) concentrations are observed across racial and ethnic groups, with higher median levels in individuals of African ancestry and lower levels in East Asian populations. Intra-group variation also exists, underscoring the need to consider both genetic structure and population background when assessing Lp(a)-related cardiovascular disease risk. Figure created in BioRender.

Unlike monogenic approaches, which focus on single, high-impact gene variations, genomics adopts a broad perspective, examining the cumulative influence of multiple genetic variations on phenotypes. Figure 3 illustrates the genetic regulation of Lp(a) synthesis and its role in CVDs.

Genetic research has significantly enhanced the understanding of CVDs through the application of GWAS and MR.^48^^,^^67^ These approaches accelerated studying the genetic basis of Lp(a) to identify genetic variants at the *LPA* gene locus that are associated with elevated Lp(a) levels, thereby linking these variants to a higher risk of CVD and CAVS.^68^

GWAS and MR studies provide valuable methodologies to address the limitations of traditional epidemiological studies. No GWAS and MR studies on Lp(a) and *LPA* have incorporated the ME population yet; however, few SNVs were identified in small studies from 4 ME countries (Table 2). The heritability of Lp(a) levels is substantial, with estimates suggesting that genetic factors account for over 50% of the variance in Lp(a) concentrations.^69^ GWAS have identified numerous SNVs associated with Lp(a) levels, which have been instrumental in elucidating the genetic architecture of lipid metabolism and its CV implications.^70^ For example, the SNV rs10455872 in the LPA gene has been identified as a significant determinant of Lp(a) concentrations, with implications for both genetic predisposition and therapeutic strategies.^71^

**Table 2:** Summary of Studies on Lp(a) Levels and *LPA* Gene Variants in the Middle Eastern Countries

Country	SNV ID	Phenotype	Population	Clinical Significance
Arab Middle Eastern Countries				
UAE^18^	NA	CAD	5,677	Approximately 15.9% of patients had abnormal Lp(a) levels (>125 nmol/L)
Lebanon/Jordan^19^	NA	CAD	617	A considerable number of patients exhibited elevated Lp(a), particularly among those on chronic statin treatment
Saudi Arabia, UAE/Lebanon^118^	NA	CAD	2,182	85.7% of patients had dyslipidemia, with a notable percentage having Lp(a) levels exceeding cardiovascular risk thresholds
Saudi Arabia^20^	NA	CAD	361	mean and median Lp(a) levels in Saudi Arabia to be 35 nmol/L and 50 nmol/L, with no influence of sex or age. Lp(a) levels were skewed toward lower values and showed minimal correlation with atherosclerotic markers and Lp(a) levels were comparable to other ethnic groups.
Jordan^119^	rs10755578 rs10455872	CAD	212/213	Significant association between the rs10455872 sequence variant and both cardiovascular disease susceptibility and moderate sensitivity to warfarin.
Iraq^120^	rs10455872	CAD	150/100	Significant association between the rs10455872-G allele and increased CAD risk
Iraq^121^	rs3798220	CAD	150/100	No significant association between rs3798220 sequence variant and CAD in the study population
Egypt^122^	NA	CAD and DM	114	Patients' lipoprotein (a) levels and Syntax scores had a highly significant positive relationship∗
Egypt^123^	NA	Acute cardiac even	314	Elevated levels of Lp(a) can help to differentiate between UA and SA patients and between UA and NSTEMI patients
Tunisia^124^	NA	DM vs healthy	300	Male patients with CHD showed significantly higher plasma Lp(a) levels than those without CHD *(P =* 0.02), and 57.3% of patients with CHD showed increase (>300 mg/L) Lp(a) compared with 33.3% of patients without CHD.
Non-Arab Middle Eastern countries				
Iran^125^	NA	Coronary heart disease (CHD)	1101	Incorporating Lp(a) into the Framingham Risk Score significantly improved risk stratification (19.6% increase in the Net Reclassification Index with better discrimination slope). Elevated Lp(a) was associated with a higher likelihood of metabolic syndrome, with each 10-unit increase corresponding to a 7% rise in odds, even after adjusting for other risk factors.
Iran^126^	rs10755578	CAD	97/94	No association between rs10755578 and increased risk for CAD
Iran^127^	rs1801693 rs7765781	Premature MI	85/85	No association between rs rs1801693 and rs7765781 and increased risk for PMI
Iran^128^	rs6415084 rs3798220	CAD	100/100	No association between the targeted sequence variants and risk of CAD
Iran^129^	rs6415084	CHD	783/783	rs6415084 was significantly associated with MI occurrence, specifically in middle-aged men
Türkiye^130^	NA	ASCVD	1193	The proportion of females with Lp(a) levels ≥90 mg/dL was higher than in males (11.4% vs. 1.4%). Lp(a) among ASCVD patients
Türkiye^115^	NA	Out-patient clinics	1,858	Lp (a) cutoff of ≥50 mg/dL was associated with CAD in both sexes, however, Lp (a) level of ≥30 mg/dL was associated with CAD only in women

SYNTAX score is a tool to risk stratify ACS patients based on anatomic features of coronary artery lesions.

NSTEMI = non-ST-segment elevation myocardial infarction, SA = stable angina; SNV ID = single nucleotide variation identifier; UA = unstable angina; UAE = United Arab Emirates; other abbreviations as in Table 1.

Moreover, the integration of MR approaches with GWAS provides insights into the causal relationships between Lp(a) and CV outcomes.^72^ For example, Wei et al^73^ utilized genetic scores, revealing that genetic variants associated with higher Lp(a) levels correlated with increased risk of coronary heart disease (CHD), independent of traditional lipid measures. This suggests that targeting Lp(a) could be a viable strategy for reducing CV risk, particularly in individuals undergoing statin therapy. GWAS has identified multiple loci associated with lipid-related traits, emphasizing the need for larger, more diverse study populations to capture the full spectrum of genetic variation.^74^

MR studies can also estimate the effect of high Lp(a), which helps in planning clinical trials that aim to lower Lp(a).^75^ The genetic determination of Lp(a) levels makes it an ideal subject for such studies, which have established a causal link between elevated Lp(a) and CVDs.^76^

The synthesis of GWAS and MR has led to the creation of polygenic risk scores, which estimate an individual's genetic susceptibility to CVD based on the cumulative effects of multiple SNVs.^77^ Two large landmark genetics studies marked a significant breakthrough in Lp(a) research.^45^^,^^78^ The first study showed a 22% higher risk of MI in those with elevated Lp(a) levels and fewer LPA K-IV2 genotypic repeats.^78^ The second study identified 2 *LPA* SNVs (rs10455872; rs 3798220) with a strong association with CHD risk among 48,742 SNVs.^45^ These SNV carriers had higher Lp(a) levels and fewer K-IV2 repeats, confirming the link between *LPA* variants and CHD.

Another important GWAS identified an *LPA* SNV substantially associated with elevated Lp(a) and the risk of CAVS.^17^ A subsequent MR study in the Danish general population showed an apparent, sequential increase in CAVS risk associated with higher Lp(a) levels and specific *LPA* risk genotypes. Individuals in the top 10% of Lp(a) concentrations had a 2- to 3-fold higher risk of CAVS, equivalent to the risk for CHD.^79^ Further studies in the same population also linked elevated Lp(a) and *LPA* genotypes to increased risks of HF, stroke, and mortality.^12^

## Ethnic Variations of Lp(a) Level

Plasma Lp(a) levels vary significantly among individuals from different racial and ethnic backgrounds.^80^ No studies from the ME highlight the ethnic variations of Lp(a) and *LPA* yet. Africans have higher Lp(a) than Caucasian or Asian populations.^81^ Studies indicate that Lp(a) concentrations are generally lower in Hispanics than Caucasians.^82^ In individuals of European descent, the distribution of Lp(a) is markedly right-skewed; for instance, in the Framingham Offspring Study, 56% of participants had Lp(a) levels between 0 to 10 mg/dL, and 70% had levels below 20 mg/dL.^83^^,^^84^ Conversely, individuals of African descent, including both Africans and African Americans, typically present Lp(a) levels that are 2 to 3 times higher than those found in Caucasians, with a distribution that more closely resembles a Gaussian curve.^85^ The relationship between elevated Lp(a) levels and CVD risk in Black populations compared with Whites remains debatable. Various studies have reported conflicting outcomes, with some indicating a higher risk while others suggest similar or even lower risk levels.^80^^,^^86^^,^^87^

**Figure 4:**
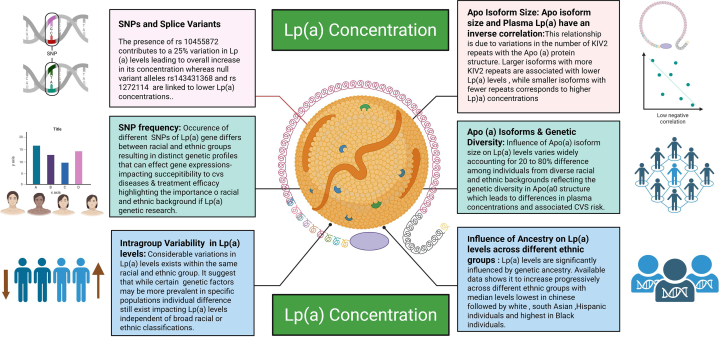
Genetic Drivers of Elevated Lipoprotein (a) and CVD Risk Genetic predisposition, particularly kringle IV type 2 (KIV-2) copy number variation and SNVs in the *LPA* gene (e.g., rs10455872, rs3798220), leads to the production of smaller Apo(a) isoforms and significantly elevated lipoprotein(a) [Lp(a)] concentrations. These concentrations exhibit high inter-individual variability influenced by inherited gene variants, genetic ancestry, and medical conditions. The liver synthesizes Apo(a), and individuals with fewer KIV-2 repeats tend to express smaller isoforms associated with higher Lp(a) levels. Elevated Lp(a) is strongly implicated in the pathogenesis of several cardiovascular diseases (CVDs), including calcific aortic valve stenosis (CAVS), coronary artery disease (CAD), heart failure (HF), and atherosclerosis, highlighting the importance of genetic screening and early risk stratification. SNVs = Single nucleotideVariations. Figure created in BioRender.

This variation is not random but is primarily attributable to genetic variations, particularly within the *LPA* gene locus. Variants within this locus are mainly responsible for the observed differences in Lp(a) levels across populations. As a result, individuals with variants may produce more or less Lp(a), resulting in the observed differences in plasma levels. Lp(a) levels, making them ideal for MR studies. Moreover, circulating Lp(a) levels serve as a reliable predictor of MACE across ethnicities, irrespective of variations in LPA SNVs or isoforms.^82^^,^^88^ Figure 4 illustrates the genetic determinants of Lp(a) levels.

The The Effect of Potentially Modifiable Risk Factors Associated with Myocardial Infarction in 52 Countries: (INTERHEART) study^89^ revealed significant ethnic disparities; specifically, Chinese individuals exhibited the lowest Lp(a) levels, while Africans had the highest, followed by South Asians. Notably, individuals with elevated Lp(a) levels (≥50 mg/dL) faced a 48% increased risk of MI. However, this association was not evident in African and Arab populations. When these groups were excluded from the analysis, the risk of AMI associated with high Lp(a) levels increased to 58%.^89^ The highest OR for MI linked to elevated Lp(a) was observed in South Asians, with an OR of 2.14 and a population attributable risk of 10%, after adjusting for age, sex, and apo (a and b).

Furthermore, a review of epidemiological and MR studies conducted within the Copenhagen population has identified Lp(a) levels of ≥20 to 30 mg/dL indicative of the atherothrombotic range.^10^^,^^78^^,^^81^^,^^83^^,^^90^^,^^91^ The findings revealed that Lp(a) levels ≥30 mg/dL, which correspond to approximately the 75th percentile in Caucasian populations, were associated with a 2- to 3-fold increased risk of CAD.^92^^,^^93^ In contrast, the European Atherosclerosis Society has established a higher threshold of ≥50 mg/dL, which aligns with the 80th percentile for European populations and the 90th percentile in the predominantly Caucasian cohort of the Framingham study.^94^ The National Heart, Lung, and Blood Institute proposed a compromise range of 30 to 50 mg for defining atherothrombotic Lp(a) levels.^93^

## Genetic Landscape of Lp(a): A Call for Research in the ME

The Arabian Peninsula has played an essential role in early human migration, serving as a crossing point for groups traveling from Africa to Eurasia and creating a genetic landscape of current world populations.^95^ Despite their common heritage, Arab populations have high genetic variability.^95^^,^^96^ The Greater Middle Eastern Variome research demonstrates this variety by finding ancient founder populations and mixing from the Gulf area, North Africa, and Central Asia.^96^^,^^97^ The genetic impact of the Arabian Peninsula is extensive across these areas, and exploring this genetic heritage is critical for understanding the variability seen in Arabs and their regional and global relationships.

Consanguinity is a deeply ingrained cultural custom in many Arab societies.^98^ This also raises the risk of recessive genetic diseases, as demonstrated by high levels of homozygosity.^97^ In the ME, which encompasses the Gulf area, North Africa, and portions of Central Asia, a staggering 63% of the genetic diseases described in the Catalogue for Transmission Genetics in Arabs (CTGA) are recessive in inheritance.^99^^,^^100^

Despite the ME populations' high consanguinity and genetic variation, they remain significantly under-represented in worldwide genomic research. Major genomic databases need more data from ME populations, limiting the application of worldwide genetic results to this area. This under-representation is significant regarding genetic differences like those seen in the *LPA* gene. Despite the high prevalence of CVDs in the ME,^99^^,^^100^ there is little data on the Lp(a) testing and genetic susceptibility associated with Lp(a) variations. In the INTERHEART study, which included 1,352 Arabs, Lp(a) levels were higher in African and Arab populations. However, the association between high Lp(a) levels (>50 mg/dL) and MI reported in other ethnic groups was not statistically significant in Africans or Arabs.^89^

Despite extensive research on *LPA* gene variations in populations from Europe, East Asia, and Africa, a notable gap exists in ME populations.^101^ A comprehensive summary of selected genetic variants in the *LPA* gene that have been extensively studied in global research or are recognized for their significant functional and clinical impacts is shown in Table 3.^46^^,^^50^^,^^82^^,^^102^, ^103^, ^104^, ^105^, ^106^, ^107^, ^108^, ^109^, ^110^, ^111^, ^112^, ^113^, ^114^, ^115^

**Table 3:** Summary of Functional and Clinically Relevant Genetic Variants in the *LPA* Gene Region in Different Populations

*LPA* Gene Variants	*LPA* Gene Region	Common Observed Effects/ References
rs76735376	Promoter	An epigenome-wide analysis identified a CpG site in the *LPA* promoter, associated with increased Lp(a) levels, overlapping the SNP rs76735376. This SNV, with a minor allele frequency of 1.1%, was linked to higher Lp (a) *(P =* 1.01× 10^−59^) and explained 3.5% of its variation. The effect is partly caused by linkage with rs10455872 and short Apo(a) isoforms.^102^^,^^103^
rs1800589	Promoter	An analysis of 5 polymorphic sites at the *LPA* gene in 27 families and unrelated individuals revealed strong linkage disequilibrium in both the 5'-region and the Kringle 4-37 sequence. Although individual sites did not directly influence Lp(a) levels, specific haplotypes were associated with certain allele sizes and concentrations, suggesting distinct expression patterns.^104^^,^^105^
rs7760010	Enhancer	Two enhancer regions upstream of the Apo(a) gene were analyzed in individuals with varying Lp(a) levels. In the DHIII region, 3 base changes were found, affecting gene expression: -1230A>G increased transcription by 2.5-fold, reporter gene activity of -1712G>T decreased by 40% This variant altered Lp(a) concentrations up to 4-fold.^107^
rs186696265	Enhancer	The SNV rs186696265, a rare variant (MAF ∼1%), showed the strongest association with Lp(a) levels and CAD risk, increasing median Lp(a) from 2.1 to 91.1 mg/dL. It also raised CAD risk by 1.73 times *(P =* 3.35 × 10^-30^).^107^
rs3124784	Protease domain	rs3124784 variant of the *LPA* gene is a frequent variation and have been shown to be associated with a 28% reduction in allelic Lp(a) expression impacting overall cardiovascular risk by potentially mitigating one of the major factors in Lp(a)-related atherogenesis.^108^
rs41267807	Protease domain	The missense variant rs41267807 in the *LPA* gene has been linked to an 89% reduction in allelic Lp(a) expression, making it a significant known genetic modifier of Lp(a) levels. GWAS shows that this variant reduces Lp(a) concentrations by approximately 5 mg/dL, indicating its significant impact on Lp(a)-related cardiovascular risk.^107^^,^^108^
rs3798220	Protease domain	The SNV rs3798220 in *LPA* gene is linked to severe CAD, with carriers having a 3.14-fold higher risk and 5-fold increased Lp(a) levels. CT and CC genotypes of this SNV also elevate OxPL/ApoB levels, enhancing Lp(a)'s atherogenic impact, especially with small apo(a) isoforms.^109^, ^110^, ^111^
rs139145675	K-V	The *LPA* variant rs39145675 is a missense variant that generates a null allele, reducing Lp(a) levels by disrupting transcription, protein folding, and secretion. This variant is associated with lower CVD risk, as it decreases Lp(a) expression and atherogenic potential, making it a protective genetic factor in CVD management.^112^^,^^113^
rs 1259144	K-IV-4	LPA missense variant rs41259144 generates null alleles caused by impaired protein folding and secretion, leading to a reduction in Lp(a) levels by 14 mg/dL in GWAS or 7 mg/dL when combined with other variants. The rs41259144 A allele lowers Lp(a) by 3.34 mg/dL, reducing CAD risk by 15% of the population's mean levels.^107^^,^^112^
rs 41270998	K-IV-5	LPA variant rs41270998 is a very rare splicing modifier located within the polypyrimidine tract, 6 bp downstream of the first exon of KIV-5. This mutation likely disrupts normal splicing, leading to reduced allelic expression of Lp(a). It is preferentially associated with smaller Lp(a) isoforms, contributing to lower plasma Lp(a) levels, which may reduce cardiovascular risk.^108^
rs 10455872	K-IV-7	LPA variant rs41267813(A) significantly lowers Lp(a) levels by interacting with rs10455872, indicating a potential protective effect. This variant explains 25% of Lp(a) variability and is linked to an increase of ≈+30 mg/dL in carriers. It is common among Caucasians but has a minor allele frequency <1% in Africans. Its association with increased *LPA* expression suggests its role in regulating Lp(a) levels and CAD risk.^50^^,^^102^^,^^110^
rs3798220	K-IV	LPA SNV rs3798220 was most prevalent in Hispanics (42.38%), rs10455872 in whites (14.27%), and rs9457951 in blacks (32.92%). The relationship to MACE is explained by elevated Lp(a) or OxPL-apoB levels.^82^^,^^114^
rs1623955	5’UTR	The variant rs1623955 is a very rare regulatory mutation in the *LPA* gene, associated with CV risk. It produces null alleles by an unknown mechanism, likely affecting Lp(a) expression. While its exact function remains unclear, it may play a role in reducing Lp(a) concentrations, potentially lowering atherosclerotic risk.^46^^,^^108^^,^^115^

Abbreviations as in Table 1.

Consequently, the findings from studies conducted in other regions may not directly apply to ME populations, which may harbor distinct *LPA* gene variations that could affect Lp(a) levels and associated CV risks. Therefore, there is a pressing need to investigate Lp(a) in the ME because the region is grappling with the growing burden of CVDs. This rising incidence and mortality are driven by a combination of genetic predispositions and lifestyle factors, alongside a concerning trend of heart attacks occurring at a younger age within this region's population.^18^

Research in this domain is crucial because it could yield population-specific insights that enhance CV risk prediction models and facilitate the approaches tailored to the genetic profiles of ME individuals. Few studies have highlighted the prevalence of elevated Lp(a) levels among the ME population, indicating a significant proportion of ME may be at increased risk for CV events.^18^, ^19^, ^20^ In the Saudi Arabian population, the mean and median Lp(a) levels in the study population were 35 and 50 nmol/L. Women often have higher Lp(a) concentrations than men. In the United Arab Emirates, around 1 in 6 patients tested for Lp(a) had abnormally high Lp(a), and CVD was prevalent in one-third of patients with high Lp(a).^18^^,^^20^

Furthermore, the genetic landscape of Lp(a) in ME populations still needs to be explored, which limits the understanding of how genetic factors contribute to CV health in these communities. By addressing this research gap, researchers can better elucidate the genetic determinants of Lp(a) levels and their implications for CVD. They could uncover population-specific genetic variants to refine CV risk prediction models and develop tailored personalized medicine approaches for the ME populations.^101^

## Level of Lp(a) and its Gene-Variation: ME Studies

The ethnic variations in Lp(a) levels and their associated risks underscore the importance of considering genetic and environmental factors.^116^ Research indicates that genetic factors significantly influence Lp(a) levels, with specific SNVs linked to ancestry-specific variations in Lp(a) concentrations.^117^ Few studies highlighted this issue across various countries in the ME (Table 2).^18^, ^19^, ^20^^,^^115^^,^^118^, ^119^, ^120^, ^121^, ^122^, ^123^, ^124^, ^125^, ^126^, ^127^, ^128^, ^129^, ^130^ However, the quality and power of these studies were not assessed. Two studies with small sample size from Egypt showed that patients with higher levels of Lp(a) had higher Syntax scores and more acute coronary events, indicating severe coronary involvement and need for interventions compared to patients with lower Lp(a) levels.^122^^,^^123^

Recent studies indicate that the prevalence of CVD in this region has escalated significantly, with CVD accounting for a substantial proportion of deaths in countries like Saudi Arabia and Lebanon, where it represented approximately 42% and 45% of total deaths, respectively.^131^ The Gulf RACE (Gulf Registry of Acute Coronary Events) and the INTERHEART analysis have highlighted that individuals in ME who experience heart attacks are typically 10 to 12 years younger than their counterparts in Western nations.^131^^,^^132^

The genetic predisposition to elevated Lp(a) levels adds complexity to CV risk management. Unlike other lipid factors that can be significantly modified through lifestyle changes or pharmacological interventions, Lp(a) levels are largely unresponsive to traditional measures. This heritability means that individuals with high Lp(a) levels often maintain these levels throughout life, making early identification crucial and mandating aggressive and targeted therapy.^14^

A study examined the association between the rs10755578 variation of the *LPA* gene and CAD in an Iranian population.^126^ There was no significant difference in the frequency of the rs10755578 variation between the case and control groups. The study concluded that the rs10755578 *LPA* variation was not associated with an increased risk for CAD.

Another study from Iran investigated the association between 2 SNVs (rs1801693 and rs7765781) in the LPA gene, previously identified by GWAS studies, and premature MI.^127^ The results showed no significant difference between the case and control groups rs1801693/rs7765781, indicating no correlation between the SNPs and premature MI.

Another Iranian study assessed the association between 2 polymorphisms in the *LPA* gene, rs6415084 and rs3798220, and the risk of CAD.^128^ It revealed no significant association between the *LPA* gene variants studied and CAD risk. The impact of polymorphisms within the *SLC22A3-LPAL2-LPA* gene cluster, which is involved in regulating Lp(a) concentrations, on CVD susceptibility was studied in Jordanian patients.^119^ Only rs10755578 SNV of the *SLC22A3-LPAL2-LPA* gene cluster (CC+CG)/GG significantly differed between cardiac patients and healthy control subjects. There was a significant association between the *SLC22A3-LPAL2-LPA* rs10455872 polymorphism and CVD susceptibility. However, other SNVs within the *SLC22A3-LPAL2-LPA* gene cluster did not significantly affect CVD.

A recent study from Iran focused on 17 SNVs within the LPA gene, exploring their relationship with the incidence of MI. The study revealed that only 1 SNP, rs6415084, exhibited a significant association with MI occurrence and the age of onset, specifically in middle-aged men.^129^

A study from Iraq explored the association between the rs10455872-G allele of the *LPA* gene and CAD in an Iraqi population.^120^ The authors reported a significant association between the rs10455872-G allele and increased CAD risk, with carriers of the AG and GG genotypes showing a higher prevalence of CAD. The minor allele frequency of the G allele was significantly higher in the patient group compared with control subjects. The study concluded that the rs10455872-G allele is a genetic risk factor for CAD, with a 2-fold increased risk for CAD in G allele carriers. Another study^121^ on the same population reported no significant association between rs3798220 polymorphism and CAD under various inheritance models. The minor allele frequency of rs3798220 also did not differ significantly between CAD and control groups, suggesting that the rs3798220 SNV is not a major genetic determinant of CAD.

## Lp(a) Genetics in the ME: How Does it Differ From Other Populations?

The association of specific SNVs in the *LPA* gene with CAD has been extensively studied across various populations, and inconsistent results have been shown.^126^, ^127^, ^128^ No studies have specifically investigated or reported the association between *LPA* gene polymorphisms and Lp(a) levels in the ME population. A meta-analysis involving 14,500 participants from 6 White European cohorts showed strong associations between *LPA* SNVs and Lp(a) levels, which correlated with CAD risk.^133^ Specifically, the study highlighted that SNVs such as rs10455872 and rs3798220 were significantly associated with increased Lp(a) levels and, consequently, higher CAD risk. A study by Clarke et al^45^ reported that the *LPA* SNVs rs10455872 and rs3798220 were strongly associated with increased Lp(a) levels, reduced copy number in the *LPA* gene, and smaller Lp(a) size .

A comprehensive analysis by Lanktree et al^134^ demonstrated that SNVs such as rs10455872 and rs3798220 are strongly associated with Lp(a) levels in South Asian, Chinese, and European Caucasian populations. Moreover, the SNV rs6415084 was associated with both K-IV-2 copy number and Lp(a) concentration and was prevalent across the 3 ethnic groups. Ugovšek et al,^58^ in an Eastern European population, identified an association between the haplotype of *LPA* gene variants rs10455872 and rs3798220 and parameters of coagulation, fibrinolysis, and inflammation in patients with MI and significantly elevated Lp(a) levels. Both genetically predicted and measured Lp(a) levels have shown associations with CAD, CAVS, and HF, even when other traditional risk factors were addressed.

It is important to note that most genetic data on the *LPA* gene have been predominantly generated from studies conducted in populations of Caucasian ancestry. Although these studies have significantly advanced the understanding of the genetic determinants of Lp(a), they leave critical gaps in the knowledge about the role of *LPA* variants in other populations, particularly in individuals of ME descent. ME population exhibits notably higher median Lp(a) levels compared to different ethnic groups globally, attributed to a higher prevalence of small Apo(a) isoforms. At the same time, Europeans show intermediate levels influenced by both small and large isoforms. South Asians tend to have elevated Lp(a) levels, contributing to their heightened CVD risk, whereas East Asians generally display lower Lp(a) levels, with larger isoforms being more common.^68^ In the ME population, limited data suggest variability in Lp(a) levels influenced by consanguinity and unique *LPA* gene variants, emphasizing the need for further research.

Genetic studies have highlighted significant links between the ME population and African ancestry.^135^ One key area of uncertainty is whether the frequency of loss-of-function (LOF) mutations in the *LPA* gene is as high in African/Middle Eastern populations as in Caucasian populations.^112^^,^^136^ LOF mutations typically result in reduced or absent apo(a) production, leading to lower circulating Lp(a) levels. If such mutations are less common in African and ME populations, this could reflect an evolutionary selective pressure in favor of higher Lp(a) levels, potentially linked to immunity. Conversely, if LOF mutations are similarly frequent, it raises the question of what counterbalances their effect, allowing for elevated Lp(a) levels in these groups. These shared genetic traits may influence the prevalence and expression of key genetic variants, including the *LPA* gene, which governs Lp(a) levels.

However, the distribution and impact of LOF mutations in the *LPA* gene remain poorly understood in both African and ME populations. Although these mutations are well-documented in populations of European descent, their frequency and functional impact in populations with African ancestry, including the ME population, are less characterized. Given the genetic overlap between ME and African populations, studying LOF mutations in the *LPA* gene within the ME context could provide valuable insights. This is particularly relevant because of the high burden of CVDs in the region and the limited data on Lp(a) genetics. Thus, exploring these ethnic differences in Lp(a) levels and LOF mutation frequencies could elucidate the evolutionary and biological significance of Lp(a), improve CVD risk stratification, and enhance the development of equitable and effective Lp(a)-targeted therapies for diverse populations.

## Guidelines on Measuring and Management of High Lp(a)

Despite the structure of Lp(a) being close to that of LDL-C, Lp(a) is more atherogenic and less frequently tested. Guidelines from the European Society of Cardiology (ESC) and Canadian Cardiovascular Society recommend Lp(a) testing for individuals with a personal or family history of premature atherosclerotic CVD or at moderate-to-high risk of CVD or at least once in a lifetime as part of primary prevention. Despite this recommendation, the Lp(a) testing rate remains lower in the ME compared with other regions. Moreover, the current data primarily focus on LDL-C and other lipid abnormalities, overlooking the role of Lp(a).^137^ A study from the USA showed that in greater than one-half million patients with atherosclerotic CVD, only 0.4% were tested for Lp(a) (a) with age and race disparities. Patients with high Lp(a) were frequently prescribed PCSK9i and ezetimibe (*P <* 0.001).^138^ There are no guidelines for testing or management of abnormal Lp(a) in the ME. Of note, Kronenberg et al^139^ summarized the consensus and guidelines on Lp(a) in Western countries and recommended early measurement and aggressive treatment of abnormal Lp(a) levels.

## Conclusions

Although Lp(a) research is progressing globally, the ME remains under-represented. Given the rapidly evolving Lp(a) research and its potential implications for cardiovascular risk assessment and management, more studies are needed on the ME populations. Such research could help clarify the prevalence of elevated Lp(a) levels, identify region-specific genetic variants, and inform targeted interventions for reducing cardiovascular risk in this population.

## Funding Support and Author Disclosures

The authors have reported that they have no relationships relevant to the contents of this paper to disclose.
